# Association of the delayed changes in glutamate levels and functional connectivity with the immediate network effects of S-ketamine

**DOI:** 10.1038/s41398-023-02346-0

**Published:** 2023-02-16

**Authors:** Lena Vera Danyeli, Zümrüt Duygu Sen, Lejla Colic, Lisa Kurzweil, Sabrina Gensberger-Reigl, Tamar Macharadze, Florian Götting, Alexander Refisch, Thomas Liebe, Tara Chand, Moritz Kretzschmar, Gerd Wagner, Nils Opel, Fabrice Jollant, Oliver Speck, Matthias H. J. Munk, Meng Li, Martin Walter

**Affiliations:** 1 Department of Psychiatry and Psychotherapy, Jena University Hospital, Jena, Germany; 2 Clinical Affective Neuroimaging Laboratory (CANLAB), Magdeburg, Germany; 3 Department of Psychiatry and Psychotherapy, University Tübingen, Tübingen, Germany; 4 Center for Intervention and Research on adaptive and maladaptive brain Circuits underlying mental health (C-I-R-C), Jena-Magdeburg-Halle, Germany; 5 German Center for Mental Health (DZPG), Site Halle-Jena-Magdeburg, Germany; 6 Food Chemistry, Department of Chemistry and Pharmacy, Friedrich-Alexander-Universität Erlangen-Nürnberg, Erlangen, Germany; 7 Department of Anesthesiology and Intensive Care Medicine, Medical Faculty, Otto-von-Guericke-Universität Magdeburg, Magdeburg, Germany; 8 Department Systems Physiology of Learning, Leibniz Institute for Neurobiology, Magdeburg, Germany; 9 Center for Behavioral Brain Sciences, Magdeburg, Germany; 10 Department of Clinical Psychology, Friedrich Schiller University, Jena, Germany; 11 School of Medicine, Université Paris-Saclay, Le Kremlin-Bicêtre, France; 12 Department of psychiatry, CHU Bicêtre, APHP, Le Kremlin-Bicêtre, France; 13 Inserm, CESP, MOODS team, Le Kremlin-Bicêtre, France; 14 Department of psychiatry, CHU Nîmes, Nîmes, France; 15 Department of Psychiatry, McGill University, Montreal, Canada; 16 Department of Behavioral Neurology, Leibniz Institute for Neurobiology, Magdeburg, Germany; 17 Department of Biomedical Magnetic Resonance, Otto von Guericke University, Magdeburg, Germany; 18 Systems Neurophysiology, Department of Biology, Darmstadt University of Technology, Darmstadt, Germany; 19 Max Planck Institute for Biological Cybernetics, Tübingen, Germany

**Keywords:** Neuroscience, Predictive markers

## Abstract

Ketamine shows rapid antidepressant effects peaking 24 h after administration. The antidepressant effects may occur through changes in glutamatergic metabolite levels and resting-state functional connectivity (rsFC) within the default mode network (DMN). A multistage drug effect of ketamine has been suggested, inducing acute effects on dysfunctional network configuration and delayed effects on homeostatic synaptic plasticity. Whether the DMN-centered delayed antidepressant-related changes are associated with the immediate changes remains unknown. Thirty-five healthy male participants (25.1 ± 4.2 years) underwent 7 T magnetic resonance spectroscopy (MRS) and resting-state functional magnetic resonance imaging (rsfMRI) before, during, and 24 h after a single S-ketamine or placebo infusion. Changes in glutamatergic measures and rsFC in the DMN node pregenual anterior cingulate cortex (pgACC) were examined. A delayed rsFC decrease of the pgACC to inferior parietal lobe (family-wise error corrected *p* (*p*_FWEc_) = 0.018) and dorsolateral prefrontal cortex (PFC; *p*_FWEc_ = 0.002) was detected that was preceded by an immediate rsFC increase of the pgACC to medial PFC (*p*_FWEc_ < 0.001) and dorsomedial PFC (*p*_FWEc_ = 0.005). Additionally, the immediate rsFC reconfigurations correlated with the delayed pgACC glutamate (Glu) level increase (*p* = 0.024) after 24 h at trend level (*p* = 0.067). Baseline measures of rsFC and MRS were furthermore associated with the magnitude of the respective delayed changes (*p*’s < 0.05). In contrast, the delayed changes were not associated with acute psychotomimetic side effects or plasma concentrations of ketamine and its metabolites. This multimodal study suggests an association between immediate S-ketamine-induced network effects and delayed brain changes at a time point relevant in its clinical context.

## Introduction

Preclinical studies showed that sub-anesthetic doses of S−/racemic ketamine, a non-competitive N-methyl-D-aspartate receptor (NMDAR) antagonist, immediately lead to changes in glutamatergic synapse properties and synaptic glutamate (Glu) levels [1–3] that in turn induce rapid synaptogenesis and a delayed antidepressant-like behavior [4, 5]. In clinical studies, the compound induces rapid antidepressant effects in individuals with major depression disorder (MDD) [6, 7]. Its antidepressant effect reaches a peak 24 h after administration [7, 8]. This antidepressant effect is preceded by psychotomimetic side effects during administration [9]. Walter et al. [10] therefore suggested a multistage drug effect of S−/racemic ketamine with both the acute changes in dysfunctional network configuration and the delayed changes in homeostatic synaptic plasticity being necessary for its antidepressant effect. While some studies reported a predictive value of immediate dissociative effects of ketamine and S-ketamine for the antidepressant response [11–14], results in the literature are inconsistent [15, 16]. Neuroimaging correlates of immediate effects are therefore worth exploring to broaden the range of the immediate effects that might be predictive of the delayed antidepressant-related changes.

Individuals with MDD show a hyperconnectivity within the default mode network (DMN), observed in resting-state functional magnetic resonance imaging (rsfMRI) studies [17, 18]. The key node of the anterior DMN, the pregenual anterior cingulate cortex (pgACC), plays an essential role in the pathophysiology of MDD [19]. Individuals with MDD also show changes in neurometabolite levels in the pgACC, measured with magnetic resonance spectroscopy (MRS). Previous studies reported reduced anterior cingulate Glu [20] or Glx levels (a combined measure of Glu and glutamine (Gln) [21–23]), which may reflect a disorder-related hypoglutamatergic state. Interestingly, Increased pretreatment pgACC activity at rest was reported to be associated with a better antidepressant response after conventional antidepressant medication, transcranial magnetic stimulation (TMS), sleep deprivation (for a review, see [24]) and racemic ketamine treatment [25, 26].

In light of MDD-related dysfunctionalities, S−/racemic ketamine-induced delayed changes in humans have been investigated at the connectivity and metabolic level. At the time point of the peak antidepressant effect, changes in resting-state functional connectivity (rsFC) in individuals with MDD have been reported, namely a reduction within the DMN 24 h after infusion [27]. In addition, these delayed rsFC changes were also observed in studies performed on healthy participants using racemic [28] as well as S-ketamine [29]. Interestingly, the S-ketamine-induced changes in the anterior cingulate cortex (ACC; pgACC and subgenual ACC) correlated with treatment outcome and clinically relevant parameters in individuals with MDD [30] and healthy controls [31], respectively. On the metabolic level, both racemic and S-ketamine induced glutamatergic modulation in individuals with MDD [32] and healthy participants [32–34]. In the pgACC specifically, an increase in Glu 24 h after S-ketamine administration correlated with a better treatment outcome [35].

Besides investigations of delayed S−/racemic ketamine effects, studies also examined the immediate functional and metabolic changes during infusion preceding its antidepressant effect. Studies on the modulation of rsFC signatures reported an increase in prefrontal connectivity of individuals suffering from MDD [36] and healthy volunteers [37] during infusion. Li et al. [28] furthermore reported an increase in resting-state fractional amplitude of low-frequency fluctuations (fALFF) in a posterior DMN region 1 h after infusion that was correlated with a delayed decrease in DMN rsFC, establishing a neural link between the acute and delayed effects in the resting-state domain. In contrast to rsFC changes, another study of our group investigated metabolic changes in the pgACC 1 h after infusion and did not observe any significant changes in glutamatergic measures [33]. While the study from Li et al. [33] acquired rather sub-acute spectral measures, a recent study investigating changes directly during infusion in healthy volunteers provided further evidence for an absence of immediate changes in Glu or Gln levels in the pgACC [38]. On the level of subjective effects, the psychotomimetic side effects during S-ketamine infusion have also been linked to changes in pgACC rsFC 24 h after administration [31].

To our best knowledge, no study investigated the association between delayed changes in rsFC and glutamatergic metabolite levels at the peak time point of the antidepressant effect of S−/racemic ketamine, 24 h after infusion, and the preceding immediate changes during infusion. In light of the evidence for the clinical relevance of the region, in this study, changes explicitly of pgACC rsFC and regional levels of glutamatergic metabolites induced by a single S-ketamine infusion were investigated using ultra-high field 7 T imaging. Walter et al. [10] suggested a multistage drug effect. Following this framework, we hypothesized that the immediate and delayed S-ketamine-induced changes within the DMN will show a positive linear association. Namely, the pgACC-centered functional and metabolic changes at the time point of the antidepressant effect may be associated with the acute rsFC changes. We furthermore explored the association of baseline functional and metabolic measures with the magnitude of delayed S-ketamine-induced changes. In a further exploratory analysis, we assessed the association of S-ketamine-induced psychotomimetic side effects and of blood plasma levels of ketamine, norketamine (NK), and hydroxynorketamine (HNK), acquired during infusion, with the neuroimaging changes.

## Materials and methods

### Study design and participants

A randomized, placebo-controlled, double-blind cross-over study was conducted to examine the association between the immediate and delayed functional connectivity and metabolic changes induced by a single sub-anesthetic S-ketamine infusion in thirty-five healthy male participants (mean age ± standard deviation (SD) = 25.1 ± 4.2 years). Since S-ketamine is approved as an add-on treatment in treatment-resistant depression (TRD), the majority of patients are already on antidepressant medication when starting S-ketamine treatment. Healthy participants were therefore chosen to allow for investigations without confounding effects of an antidepressant treatment. Furthermore, since both immediate [37, 38] and delayed [28, 29] brain changes in healthy participants have been already reported in the literature, we expected to observe the hypothesized relationship also in healthy participants. Indeed, a previous study performed in healthy participants showed the association between the effects 1 h and 24 h after infusion [28].

The main inclusion criteria were: no current or lifetime major psychiatric disorder, including substance or alcohol dependence or abuse, according to DSM-IV [39] as assessed by the Structured Clinical Interview for DSM-IV (SCID) [40]; no family history of psychiatric disorders as assessed by a demographic questionnaire and no neurological or physical constraints or severe illnesses as evaluated by a study physician during screening. Further inclusion criteria were right-handedness and the absence of magnetic resonance imaging (MRI) contraindications. All participants gave informed written consent. The study was reviewed and approved by the Institutional Review Board of the Otto-von-Guericke-University Magdeburg and was performed in accordance with the recommendations made in the Declaration of Helsinki and local legal requirements. This is the first analysis performed and reported on this study and data obtained from this study has not been used for other publications before.

### Experimental procedure

**Fig. 1:**
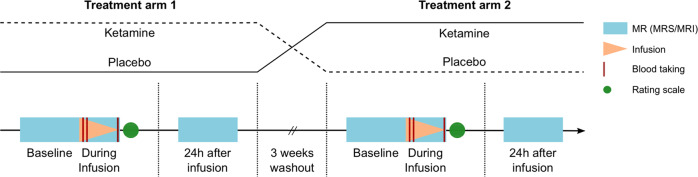
Study design. The experimental procedure consisted of two consecutive days per treatment arm. On day 1 (infusion day), participants underwent MR scanning before and during S-ketamine or placebo infusion. Blood samples were obtained 8, 13 and 48 min after the beginning of the infusion. On day 2 (24 h after infusion), MR scanning was repeated with the same neuroimaging measurements. Additional HR rsfMRI was acquired on all measurement days, unrelated to this analysis. Treatments were crossed-over with a washout period of 3 weeks on average. HR high-resolution, MR magnetic resonance, MRS magnetic resonance spectroscopy, rsfMRI resting-state functional magnetic resonance imaging.

The experimental procedure consisted of two consecutive days per treatment arm, including magnetic resonance (MR) scanning before infusion (for baseline measures), during infusion (for immediate effects), and 24 h after infusion (for delayed effects), as well as blood sampling during infusion (Fig. 1). The interval between the two treatment arms was approximately 3 weeks to avoid carry-over effects. Approximately 2 weeks after each infusion, a short follow-up visit with blood sampling and a check-up with a physician were conducted, unrelated to the present analysis.

Due to its higher affinity for NMDAR, S-ketamine has been the focus of research, and the intranasal form of the S-isomer (Spravato®) [41] was approved by the FDA for TRD. Participants received a single intravenous (i.v.) infusion (Injectomat® MC Agilia; Fresenius Kabi GmbH, Bad Homburg, Germany) of S-ketamine hydrochloride (0.33 mg/kg body weight; “Ketanest S Pfizer”) or 0.9% saline as the placebo control in a randomized order. The infusion consisted of a bolus (0.11 mg/kg body weight S-Ketamine or 0.9% saline) administered over 8 min followed by a maintenance dose (0.22 mg/kg body weight S-Ketamine or 0.9% saline) administered over 40 min with a 2 min break between the bolus and the maintenance infusion. Since S-ketamine was administered, the dosage was reduced compared to the standard infusion regime of racemic ketamine of 0.5 mg/kg [42] while ensuring a psychoactive dose that was well-tolerated inside the MR setup. We aimed to reach an antidepressant effect-relevant drug level between 70 ng/ml and 200 ng/ml [43, 44] during maintenance infusion and desired to perform the imaging measurements when ketamine plasma levels showed a relative steady-state. For this reason, a bolus was used to reach the desired concentration as fast as possible. An infusion protocol with a bolus duration of 8 min was found to fit our study design best, as assessed by a 3-compartment pharmacokinetic model (Tivatrainer V9.1; www.eurosiva.com) and was hence employed in the present study. The 2 min break was included to ensure tolerability of the combination of the initial bolus and the maintenance infusion. Vital signs were monitored throughout the infusion (NONIN Pulse Oximeter 8600-FO), and participants were observed for a minimum of 4 h after infusion to ensure medical safety.

To quantify the extent of the psychotomimetic side effects, participants completed the 5-Dimensional Altered States of Consciousness Rating Scale (5D-ASC; [45]) retrospectively after each infusion. The 5D-ASC was chosen since it is a self-report questionnaire. Based on our DMN-centered hypothesis, we assumed that a self-report scale would reflect the very transient acute effect in a more sensitive way than e.g., the often used Clinician-Administered Dissociative States Scale (CADSS; [46]). Additional behavioral tasks, electroencephalography (EEG), and further questionnaires were obtained as part of the study protocol, yet unrelated to the present analysis.

### Magnetic resonance data acquisition

MR images were acquired on an ultra-high field 7 T scanner (Siemens Healthineers, Erlangen, Germany) with a 32–channel head array coil. Participants underwent structural MRI before and 24 h after infusion and MRS and rsfMRI before, during, and 24 h after infusion in a fixed order. During infusion, MRS data acquisition started with the beginning of the maintenance infusion, while the subsequent rsfMRI data was acquired approximately 36 min after the beginning of the infusion (mean ± SD: 36.18 ± 2.34 min). Since the acute rsFC was the primary measure of interest in this study compared to acute metabolic measures, the rsfMRI data was acquired at the second half of the infusion, the time interval when ketamine plasma levels were expected to show the most stable steady-state.

For T1 acquisition: After automated shimming, a T1-weighted structural MR image was obtained using a magnetization-prepared rapid gradient-echo (MPRAGE) sequence with the following parameters: echo time (TE) = 2.54 ms, repetition time (TR) = 1700 ms, inversion time (TI) = 1050 ms, flip angle = 5 °, field of view (FoV )= 256 mm, 176 slices, grappa acceleration factor PE = 2, bandwidth = 160 Hz/pixel, isotropic voxel size = 1 mm^3^.

**Fig. 2:**
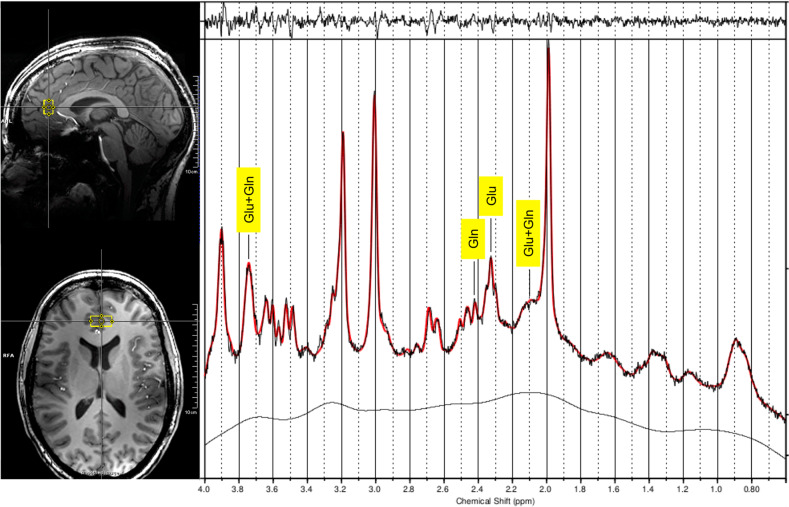
Example voxel positioning in the pgACC and fitted spectral curve using LCModel. As reported in Dou et al. [47], the pgACC voxel was positioned touching the genu of the corpus callosum while bypassing the callosomarginal artery. The bottom of the voxel was tilted into the anterior commissure-posterior-commissure plane and oriented using the sagittal projection line. Gln glutamine, Glu glutamate, MRS magnetic resonance spectroscopy, pgACC pregenual anterior cingulate cortex.

For MRS acquisition: After a region-specific automated shimming, a stimulated-echo acquisition mode (STEAM) sequence was employed to measure the spectra within the pgACC with the following parameters: voxel size = 20 × 15 × 10 mm^3^, TE = 20 ms, TR = 3000 ms, mixing time (TM) = 10 ms, bandwidth = 2800 Hz, 128 averages. Water signal with a single average served as an internal reference for quantification and eddy current correction. The positioning of the pgACC MRS voxel followed anatomical landmarks [47]. Furthermore, spectra of the anterior midcingulate cortex (aMCC) and dorsal posterior cingulate cortex (dPCC) were obtained (before and 24 h after infusion only), unrelated to the present analysis. The acquisition of the different MRS voxels was performed in a randomized order and lasted on average 11 min per voxel. Neurometabolites of interest were Glu and Gln (gamma-aminobutyric acid (GABA) was analyzed in an exploratory manner that was not directed towards answering our primary research question, for details, see Supplementary Information). An example of voxel positioning and spectrum is displayed in Fig. 2. MRS data preprocessing and metabolite fitting are reported in detail in the Supplementary Information.

For the eyes-closed rsfMRI scans: An echo-planar imaging (EPI) sequence was applied with the following parameters: TE = 25 ms, TR = 1500 ms, flip angle = 70 °, FoV = 212 mm, 60 slices, isotropic voxel size = 2 mm^3^, multi-band acceleration factor = 3, grappa acceleration factor PE = 2. Before the EPI sequence, field maps were acquired for susceptibility distortion correction of the EPI with a dual-echo gradient-echo sequence with the following parameters: TE1 = 6 ms, TE2 = 7.02 ms, TR = 1200 ms, flip angle = 60 °, FoV = 212 mm, slice thickness = 2 mm, bandwidth = 259 Hz/pixel. The duration of the rsfMRI scans for the three time points was, on average, 11 min (average of 438 volumes) with slight variations due to the infusion procedural requirements, while the number of volumes did not significantly differ between the treatment arms (*p* > 0.05).

### Structural and functional magnetic resonance imaging data preprocessing and functional connectivity calculation

Structural and functional MR data preprocessing was performed employing fMRIPrep 20.1.1 ([48]; RRID:SCR_016216), which is based on Nipype 1.5.0 ([49]; RRID:SCR_002502), together with FMRIB’s ICA-based Xnoisifier (FIX; [50, 51]) for noise removal in the functional MR data. Preprocessing steps are reported in detail in the Supplementary Information. The MRS pgACC voxel mask (40% voxel overlap between participants across all baseline runs) was used as a seed for the rsFC analysis. The 40% overlap was a result of the challenges in individual voxel placement in the pgACC region (for details, see Supplementary Information and Supplementary Fig. S1). RsFC maps were calculated by Pearson correlation, and Fisher Z transformed using xcpEngine.

### Blood sampling and analysis of plasma levels of ketamine and ketamine metabolites

Venous blood samples were collected from an arm vein at 8, 13, and 48 min after the beginning of the infusion. Ketamine (KET) and ketamine metabolite (norketamine (NK) and hydroxynorketamine (HNK)) plasma levels (KET8, KET13, KET48, NK8, NK13, NK48, HNK8, HNK13, HNK48) were measured. Blood samples were preprocessed and analyzed with liquid chromatography hyphenated targeted mass spectrometry using enantiomer-unspecific standards for the simultaneous analysis of ketamine, NK, and HNK (for details, see [52]).

### Statistical analysis

The statistical analysis was adopted from a similar experimental design by Scheidegger et al. [29]. While our primary aim was to investigate a potential correlation between the S-ketamine-induced changes compared to baseline of the two investigated time points during infusion and 24 h after infusion, LME models for the immediate and the delayed effects were performed (for details, see Supplementary Information and Supplementary Figs. S2, S3). To obtain the changes from baseline at the investigated time points for the correlational analysis, paired t-tests were performed separately for each treatment arm (S-ketamine and placebo) to assess delayed (24 h after infusion vs. baseline) changes in the pgACC glutamatergic metabolite levels (Glu, Gln, Gln/Glu) and rsFC, as well as immediate changes (during infusion vs. baseline). For the primary outcome—the correlation between the delayed functional and glutamatergic changes and the immediate rsFC changes—the rsFC time courses were extracted (4 mm radius sphere around the peak voxel) from regions that showed significant rsFC changes. Delta rsFC values were calculated for the immediate (during infusion-baseline) and delayed (24 h after infusion-baseline) changes. In an exploratory manner, the association of the delayed Glu level and rsFC changes with their respective baselines was investigated. Based on Oldham [53], the following pairs were correlated: (b-a) and (a + b)/2 (b = baseline and a = 24 h after infusion; hereafter referred to as adjusted baseline) to avoid the problem of mathematical coupling. For further exploratory analyses, associations of the significant delayed rsFC and metabolic changes with S-ketamine-induced psychotomimetic effects (as assessed by the 5D-ASC; [45]) and with plasma levels of ketamine, NK, and HNK were examined. Due to the low number of available data for both NK and HNK at 8 and 13 min after the start of the infusion, only NK48 and HNK48 were used for the correlation analyses (acute imaging changes were correlated with the psychotomimetic effects and ketamine metabolite plasma levels in an additional analysis reported in the Supplementary Information).

Paired t-tests conducted within the treatment arm were corrected for multiple comparisons of the other arm (adjusted *α* = 0.025). Time-point comparisons of the whole-brain level rsFC were performed in SPM12 (http://www.fil.ion.ucl.ac.uk/spm/), and the differences were considered significant at a cluster-level family-wise error (FWE) corrected threshold, *p*_FWEc_ < 0.025 with an initial single voxel threshold, *p* < 0.001. For correlation analyses, statistical thresholds were set at *p* < 0.05, corrected for multiple comparisons applying Bonferroni’s correction where indicated. The significance value was obtained by a one-tailed test when hypothesis-driven, namely the correlational analysis between time points since a positive correlation was expected, and a two-tailed test when exploratory (all other analyses). Meanwhile, Pearson correlations were performed when data were normally distributed (correlation coefficient expressed as *r*_p_); otherwise, Spearman nonparametric correlations were applied (correlation coefficient expressed as *r*_s_). Statistics were performed, and figures were generated using R (version 4.0.3) with the package ggpubr (version 0.4.0) and Matlab toolbox BrainNet Viewer (version 1.7).

## Results

### Data characteristics

After excluding one participant due to insufficient data quality, 34 participants were included in all rsFC analyses. MRS quality was determined for every metabolite and run separately. Therefore, the number of spectra included in the MRS analysis differed between metabolites, treatment arms, and time points (see Supplementary Table S1). Plasma levels of ketamine, NK, and HNK during infusion and the number of available data points are reported in detail in Supplementary Table S2.

### Immediate effects of S-ketamine on pgACC whole-brain rsFC and its regional levels of glutamatergic metabolites

**Fig. 3:**
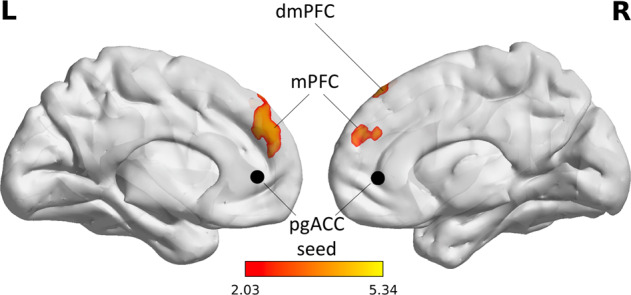
PgACC resting-state functional connectivity during S-ketamine infusion. PgACC rsFC increases during infusion in the S-ketamine arm. MPFC (*x* = 2, *y* = 56, *z* = 24, *k* = 924, Bonferroni adjusted *α* = 0.025, *p*_FWEc_ < 0.001) and dmPFC (*x* = 40, *y* = 38, *z* = 52, *k* = 338, Bonferroni adjusted *α* = 0.025, *p*_FWEc_ = 0.005). dmPFC dorsomedial prefrontal cortex, mPFC medial prefrontal cortex, pgACC pregenual anterior cingulate cortex, rsFC resting-state functional connectivity.

A paired-sample t-test revealed an immediate significant increase in rsFC (during infusion>baseline) from the pgACC to medial prefrontal cortex (mPFC; *x* = 2, *y* = 56, *z* = 24, cluster size (*k*) = 924, adjusted *α* = 0.025, *p*_FWEc_ < 0.001) and dorsomedial prefrontal cortex (dmPFC; *x* = 14, *y* = 38, *z* = 52, *k* = 338, adjusted *α* = 0.025, *p*_FWEc_ = 0.005) in the S-ketamine arm (Fig. 3 and Supplementary Table S3). There were no significant findings in the other contrast (baseline>during infusion) or the placebo arm.

For the metabolite analysis, the minimal detectable concentration differences in our data were estimated as 0.39 μmol/g (~7%) for Glu and 0.4 μmol/g (~17%) for Gln, respectively. Even though Glu changes were within the detectable range, no significant immediate change in Glu levels in either treatment arm was revealed. For Gln, changes below the minimum detectable concentration difference were observed. Therefore, the analysis at both investigated time points of Gln and Gln/Glu was performed as exploratory. Same as for acute Glu changes, no significant changes in the Gln level or Gln/Glu ratio in either treatment arm were observed (for metabolite levels, see Supplementary Table S1).

### Delayed effects of S-ketamine on pgACC whole-brain rsFC and its regional levels of glutamatergic metabolites

**Fig. 4:**
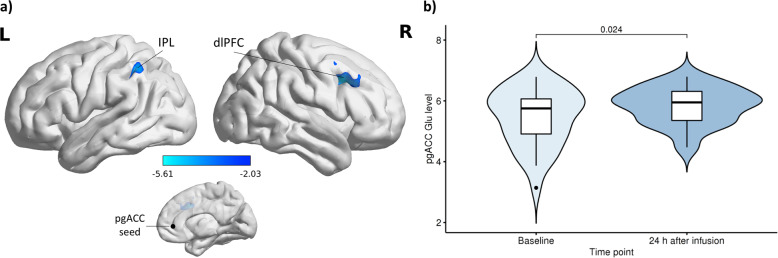
PgACC resting-state functional connectivity and glutamate levels 24 h after S-ketamine infusion. PgACC rsFC decreases and Glu level increases 24 h after infusion in the S-ketamine arm. **a** Left IPL (*x* = −44, *y* = −42, *z* = 38, *k* = 239, Bonferroni adjusted *α* = 0.025, *p*_FWEc_ = 0.018), dlPFC (*x* = 40, *y* = 16, *z* = 38, *k* = 368, Bonferroni adjusted *α* = 0.025, *p*_FWEc_ = 0.002) and cerebellum (*x* = 34, *y* = −60, z = −32, *k* = 250, Bonferroni adjusted *α* = 0.025, *p*_FWEc_ = 0.015, not shown here) (pgACC seed: black sphere). **b** PgACC Glu levels showed a significant increase 24 h after infusion compared to baseline in the S-ketamine group (*n* = 32, *t*(31) = −2.36, Bonferroni adjusted *α* = 0.025, *p* = 0.024). dlPFC dorsolateral prefrontal cortex, Glu glutamate, IPL inferior parietal lobe, pgACC pregenual anterior cingulate cortex, rsFC resting-state functional connectivity.

A significant decrease in pgACC rsFC (baseline>24 h after infusion) was observed in the left inferior parietal lobe (IPL; *x* = −44, *y* = −42, *z* = 38, *k* = 239, adjusted *α* = 0.025, *p*_FWEc_ = 0.018), right dorsolateral prefrontal cortex (dlPFC; *x* = 40, *y* = 16, *z* = 38, *k* = 368, adjusted *α* = 0.025, *p*_FWEc_ = 0.002) and cerebellum (*x* = 34, *y* = −60, *z* = −32, *k* = 250, adjusted *α* = 0.025, *p*_FWEc_ = 0.015) in the S-ketamine arm (Fig. 4a and Supplementary Table S3). There were no significant findings in the other contrast (24 h after infusion>baseline) or the placebo arm.

A significant Glu increase 24 h after infusion was detected in the S-ketamine arm only (*t*(31) = −2.36, *α* = 0.025, *p* = 0.024) (Fig. 4b), while no significant changes in the Gln or Gln/Glu levels in either treatment arm were observed in the exploratory analysis (for metabolite levels, see Supplementary Table S1).

### Association between the delayed changes and the immediate S-ketamine-induced effects

**Fig. 5:**
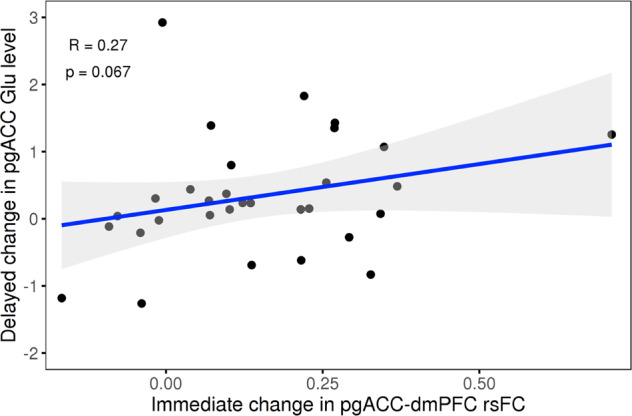
Correlation between immediate and delayed measures in the pgACC in the S-ketamine arm. A trend-level positive correlation of the immediate rsFC increase (during infusion-baseline) between the pgACC and dmPFC and the delayed pgACC Glu increase (24 h after infusion-baseline) (*n* = 31, *r*_p_ = 0.27, Bonferroni adjusted (for dependent variables) *α* = 0.04, *p* = 0.067) was observed. The shaded area represents the 95% confidence interval of the fit. dmPFC dorsomedial prefrontal cortex, Glu glutamate, pgACC pregenual anterior cingulate cortex, rsFC resting-state functional connectivity.

No significant correlations between the immediate and the delayed rsFC changes were detected. Between the modalities, a trend-level significant correlation between the immediate increase in pgACC-dmPFC rsFC and the delayed increase in Glu levels was observed (adjusted *α* = 0.04, with Bonferroni correction for dependent variables (www.quantitativeskills.com), *r*_p_ = 0.274, *p* = 0.067; Fig. 5).

### Association of delayed S-ketamine-induced changes with respective baseline measures

Exploratory analysis examining the baseline association of the observed delayed brain changes revealed a significant negative correlation between the delayed decrease in pgACC-left IPL rsFC and its respective baseline (*r*_p_ = −0.465, *α* = 0.05, *p* = 0.0053) (Supplementary Fig. S4a). No other significant correlations were observed for the delayed rsFC changes. Furthermore, a significant negative correlation between the delayed Glu level increase with its baseline was found in the S-ketamine treatment arm (*r*_s_ = −0.500, *α* = 0. 05, *p* = 0.004) (Supplementary Fig. S4b).

### Association of delayed S-ketamine-induced changes with psychotomimetic side effects during S-ketamine infusion

As assessed by the 5D-ASC [45], S-ketamine infusion (mean scores of the main scales in the S-ketamine arm: oceanic boundlessness = 86.15; anxious ego dissolution = 32.46; visual restructuralization = 58.61; auditory alterations = 25.27; and reduction of vigilance = 42.08) induced a robust acute psychotomimetic effect compared to the placebo infusion (mean scores of the main scales in the placebo arm: oceanic boundlessness = 3.21; anxious ego dissolution = 4.6; visual restructuralization = 1.69; auditory alterations = 2.91; and reduction of vigilance = 11.62) (*n* = 35; *p*’s<0.001). Correlation analysis revealed no significant association between the main scales of the questionnaire and the delayed S-ketamine-induced brain changes (both Glu levels and rsFC). Furthermore, an additional analysis beside the primary study interest, correlating the acute imaging changes with the psychotomimetic side effects, also did not yield any significant correlations (for details, see Supplementary Information).

### Association of delayed S-ketamine-induced changes with plasma levels of ketamine and its metabolites

No significant correlation between the delayed neuroimaging measures (both Glu levels and rsFC) and ketamine, NK, and HNK plasma levels was observed in this exploratory investigation. A trend-level significant positive correlation between the pgACC-Ieft IPL rsFC 24 h after infusion and the ketamine plasma level at 48 min after the start of the infusion (KET48) was observed (*r*_p_= 0.326, *α* = 0.05, *p* = 0.06) (Supplementary Fig. S5). No other correlations were found. Additionally, an analysis beside the primary study interest, correlating the acute imaging changes with the plasma levels, also did not reveal any significant correlations (for details, see Supplementary Information).

## Discussion

In this multimodal ultra-high field MR imaging study in healthy male participants, we investigated the association between the pgACC-centered S-ketamine-induced immediate rsFC changes and the delayed functional and metabolic changes 24 h after infusion. A single S-ketamine infusion induced a rsFC reduction within the DMN 24 h after infusion, which was preceded by an immediate increase in within DMN rsFC. Contrary to our hypothesis, there was no significant association between immediate and delayed rsFC changes. While, as expected, pgACC Glu levels during infusion did not significantly change, a delayed Glu level increase was observed. This delayed increase in pgACC Glu levels approached a trend-level correlation with the immediate increase in the rsFC between the pgACC and dmPFC. Furthermore, higher baseline measures of rsFC between nodes of the DMN were associated with the magnitude of the delayed rsFC decrease; similarly, lower baseline Glu levels were linked to a more pronounced delayed S-ketamine-induced increase in Glu levels. Psychotomimetic side effects or plasma levels of ketamine and its metabolites during infusion were not significantly associated with the S-ketamine-induced brain changes.

To separately interpret the observed delayed changes in both modalities, rsFC first MRS second, they will be briefly elaborated on in the following paragraph. We observed a delayed rsFC decrease between the pgACC and IPL. This decrease in rsFC within the DMN is in line with previous evidence from multiple studies [28, 29, 54] and has been associated with better antidepressant treatment response to conventional antidepressant medication [55]. Aberrant rsFC within the DMN has been suggested as a trait biomarker of MDD and may be a potential therapeutic target [56, 57]. While our findings cannot be directly translated to clinical investigations, reduced connectivity within the DMN could indicate a normalization of depression-related connectivity patterns.

The detected rsFC decrease between regions of the two resting-state networks DMN (pgACC) and central executive network (CEN) (dlPFC) 24 h after infusion is of additional interest. The CEN is involved in cognitive control and externally focused attention [58, 59], which are impaired in MDD [60, 61]. Racemic and S-ketamine were found to decrease cognitive symptoms [62] and increase cognitive performance [63, 64]. Furthermore, in individuals with depression, the DMN shows a relative dominance over the CEN, with a stronger effective connectivity from the DMN to CEN than vice versa [65]. A ketamine-induced decrease of the rsFC between the DMN and CEN may therefore reestablish the balance between the two resting-state networks. The relief in cognitive symptoms could be related to an enhanced prefrontal control [30] that may be mediated by the proposed rapid synaptogenesis [66]. However, the directionality of associations between pro-plastic effects and those on between-network connectivity remains hard to interpret. This results from the, probably polysynaptic, complexity of the mechanisms underlying observable correlation of spontaneous fMRI time series, which ideally will be targeted directly using appropriate imaging studies in animals.

Regarding S-ketamine-induced changes after 24 h at the metabolic level, we detected a significant delayed increase in pgACC Glu levels. This finding is in line with studies reporting delayed prefrontal glutamatergic modulation in individuals with MDD [67] and healthy participants [33, 34, 67], as well as with a recent study reporting increased Glu levels in the pgACC 24 h after S-ketamine administration [35]. Since Gln is the non-neuroactive precursor and metabolite in the Glu–Gln cycle and has been suggested as a marker of excitatory neurotransmission [68, 69], it may also serve as a sensitive indicator for acute downstream effects following S−/ketamine administration. While a change in both metabolites, Glu and Gln, was expected, the lack of a change in the Gln level can be interpreted in the way that it is a result of the minimal detectable concentration difference calculated for Gln in the present dataset.

In the following paragraph, we will shed light on the immediate S-ketamine-induced changes in both imaging modalities, first the rsFC followed by the MRS, as well as on behavioral changes and ketamine blood levels. An immediate rsFC increase has been reported between the pgACC and mPFC/dmPFC, which bears an interesting parallel to studies reporting an increase in within DMN connectivity [70], and increased rsFC within the PFC during infusion in healthy participants [37]. The antidepressant-like behavior 24 h after racemic ketamine administration has been linked to the rapid (within hours) increase in synaptic number and function in the PFC [5, 71–73]. This acute synaptic reconfiguration could serve to reverse prefrontal cortical synapse loss observed in multiple models of MDD and thereby reinstate PFC functionality [74, 75]. The here reported immediate effects might reflect the initiation phase of these acute changes at the synaptic level observed in the PFC in animal studies. Even though acute synaptic changes might be related to dissociative side effects in other brain regions that are more involved in dissociation [76, 77], our observed pgACC-seeded rsFC reconfiguration could not be explained by the prominent psychotomimetic side effects of S-ketamine.

During infusion, on the neurometabolic level, no glutamatergic changes were observed, as expected for the investigated region. The lack of immediate glutamatergic modulation is consistent with a recent study that did not find significant changes in Glu, Gln, or Gln/Glu ratio in the pgACC during S-ketamine infusion [38]. This may be due to a very transient increase in Glu that was not captured by our measurement.

Investigating the effects with respect to our primary hypothesis, the association of immediate and delayed changes, we did not see a significant link between the two time points in the rsFC domain. While the acute psychotomimetic side effects also were not associated with the delayed brain changes, the immediate increase in prefrontal connectivity was associated with the delayed Glu changes. It can be put forward that the increased rsFC during S-ketamine infusion serves as an initial disruption of the functional network that is dysfunctional in MDD, thereby enabling reconfiguration in the pgACC 24 h after infusion, which might have presented itself with an increase in Glu levels.

Examining the association between the baseline measurements and S-ketamine-induced imaging changes, our exploratory results showed a negative correlation between the baseline rsFC measures and the delayed reduction of rsFC (pgACC-IPL), and between the baseline MRS Glu levels and the delayed Glu increase. Therefore, it can be assumed that higher rsFC baseline values in regions of the DMN, as previously observed in individuals with MDD [17, 18], are associated with stronger S-ketamine-induced changes, whereas lower glutamatergic levels at baseline, as found in individuals with MDD [20–23], are associated with a larger increase in Glu 24 h after infusion. Even though our sample consisted of healthy participants, this finding suggests that participants with a neuroimaging “biotype” closer to the neuroimaging characteristics observed in individuals with MDD show larger rsFC and Glu level changes following S-ketamine.

Due to S−/racemic ketamine’s short half-life and the delayed occurrence of the antidepressant-related effects, its metabolites gained attention for their roles in the mechanism of action following administration [44, 78, 79]. Here, neither ketamine plasma levels nor plasma levels of its metabolites during infusion showed a significant association with the observed imaging changes. Of note, a trend-level correlation of the delayed pgACC-left IPL rsFC change with the ketamine plasma level 48 min after the start of infusion was observed. Existing preliminary studies reported inconclusive observations regarding the associations of plasma levels with the investigated neurometabolite levels [80, 81]. Therefore, the link between peripheral plasma levels during infusion and the cascade of central nervous system changes following administration warrants further investigations.

### Limitations

Several limitations have to be acknowledged that further work may address. First, it is worth mentioning that, while our infusion protocol does not directly follow the protocols commonly applied in the clinical context during the treatment of patients, it was designed for the specific use of the neuroimaging investigation during a steady-state. This was important on the methodological level to answer our research question; therefore the mechanistic approach of this study differs from the clinical application of ketamine. Second, although investigations in healthy participants yield clinical relevance and congruent observation in healthy individuals and individuals with MDD were made [82], a further limitation is the lack of comparison with individuals with MDD. The presented findings in healthy participants therefore serve as an initial investigation of the link between the immediate and delayed changes after S-ketamine. Third, in the present study, it may be plausible that the time points of blood sampling during infusion were too early to achieve comparable levels of ketamine in the plasma across participants. We also suggest that NK and HNK should be investigated at later time points since their concentrations were often below the limit of detection or quantification, respectively, at the beginning of the infusion. Furthermore, in future studies it would also be worth investigating S-ketamine and its metabolites in an enantiomer specific approach to get more precise measures specifically for the roles of S-ketamine and its metabolites in the mechanisms of action.

## Conclusion

In conclusion, we found that a single S-ketamine infusion induces a delayed decrease in rsFC of the DMN, which is preceded by an immediate DMN rsFC increase. The immediate change in rsFC was linked to a delayed Glu level increase in the pgACC, revealing a cross-modal association between the investigated time points. Moreover, baseline measures were associated with the magnitude of the S-ketamine-induced changes. Future studies should assess baseline characteristics as potential markers for treatment response in individuals with MDD. Therefore, our study highlights a complex interplay of network and metabolic activity within the first 24 h following S-ketamine administration.

## Supplementary information

Supplementary Information
