# Correction: Association of the delayed changes in glutamate levels and functional connectivity with the immediate network effects of S-ketamine

**DOI:** 10.1038/s41398-023-02377-7

**Published:** 2023-02-28

**Authors:** Lena Vera Danyeli, Zümrüt Duygu Sen, Lejla Colic, Lisa Kurzweil, Sabrina Gensberger-Reigl, Tamar Macharadze, Florian Götting, Alexander Refisch, Thomas Liebe, Tara Chand, Moritz Kretzschmar, Gerd Wagner, Nils Opel, Fabrice Jollant, Oliver Speck, Matthias H. J. Munk, Meng Li, Martin Walter

**Affiliations:** 1 Department of Psychiatry and Psychotherapy, Jena University Hospital, Jena, Germany; 2 Clinical Affective Neuroimaging Laboratory (CANLAB), Magdeburg, Germany; 3 Department of Psychiatry and Psychotherapy, University Tübingen, Tübingen, Germany; 4 Center for Intervention and Research on adaptive and maladaptive brain Circuits underlying mental health (C-I-R-C), Jena-Magdeburg-Halle, Germany; 5 German Center for Mental Health (DZPG), Site Halle-Jena-Magdeburg, Germany; 6 Food Chemistry, Department of Chemistry and Pharmacy, Friedrich-Alexander-Universität Erlangen-Nürnberg, Erlangen, Germany; 7 Department of Anesthesiology and Intensive Care Medicine, Medical Faculty, Otto-von-Guericke-Universität Magdeburg, Magdeburg, Germany; 8 Department Systems Physiology of Learning, Leibniz Institute for Neurobiology, Magdeburg, Germany; 9 Center for Behavioral Brain Sciences, Magdeburg, Germany; 10 Department of Clinical Psychology, Friedrich Schiller University, Jena, Germany; 11 School of Medicine, Université Paris-Saclay, Le Kremlin-Bicêtre, France; 12 Department of psychiatry, CHU Bicêtre, APHP, Le Kremlin-Bicêtre, France; 13 Inserm, CESP, MOODS team, Le Kremlin-Bicêtre, France; 14 Department of psychiatry, CHU Nîmes, Nîmes, France; 15 Department of Psychiatry, McGill University, Montreal, Canada; 16 Department of Behavioral Neurology, Leibniz Institute for Neurobiology, Magdeburg, Germany; 17 Department of Biomedical Magnetic Resonance, Otto von Guericke University, Magdeburg, Germany; 18 Systems Neurophysiology, Department of Biology, Darmstadt University of Technology, Darmstadt, Germany; 19 Max Planck Institute for Biological Cybernetics, Tübingen, Germany

**Keywords:** Neuroscience, Predictive markers

Correction to: *Translational Psychiatry* 10.1038/s41398-023-02346-0, published online 16 February 2023

The original version of this article contained a mistake. The authors noticed that one document, the co-author agreement, was also published as supplementary information. This document contains email correspondence for the agreement of including an additional co-author but is not a document related to the manuscript. The authors therefore want to remove this document from the SI since it is irrelevant for outside readers. The original article has been corrected.

