# Supplementary material for: Association of the delayed changes in glutamate levels and functional connectivity with the immediate network effects of S-ketamine
Source: Transl Psychiatry. 2023 Feb 16;13:60. doi: 10.1038/s41398-023-02346-0 (PMC9935558; doi:10.1038/s41398-023-02346-0)
Supplement: Supplementary file 1 — Supplementary Information [file 41398_2023_2346_MOESM1_ESM.docx]

**Supplementary Information „Association of the delayed changes in glutamate levels and functional connectivity with the immediate network effects of S-ketamine“**

Lena Vera Danyeli, MSc^1,2,3^, Zümrüt Duygu Sen, MD PhD^1,2,3,4^, Lejla Colic, PhD^1,2,4,5^, Lisa Kurzweil^6^, Sabrina Gensberger-Reigl, PhD^6^, Tamar Macharadze, MD^7,8,9^, Florian Götting, MD^1,2^, Alexander Refisch, MD^1^, Thomas Liebe, MD^1,2^, Tara Chand, MSc^1,2,3,10^, Moritz Kretzschmar, MD, PhD^7^, Gerd Wagner, PhD^1,4^, Nils Opel, MD^1,4,5^, Fabrice Jollant, MD PhD^1,11,12,13,14,15^, Oliver Speck PhD^4,5,9,16,17^, Matthias H.J. Munk, MD^3,18^, Meng Li, PhD^1,2,4^*, Martin Walter, MD PhD^1,2,3,4,5,9,16,19^*

1 Department of Psychiatry and Psychotherapy, Jena University Hospital, Jena, Germany

2 Clinical Affective Neuroimaging Laboratory (CANLAB), Magdeburg, Germany

3 Department of Psychiatry and Psychotherapy, University Tübingen, Tübingen, Germany

4 Center for Intervention and Research on adaptive and maladaptive brain Circuits underlying mental health (C-I-R-C), Jena-Magdeburg-Halle, Germany

5 German Center for Mental Health (DZPG), Site Halle-Jena-Magdeburg, Germany

6 Food Chemistry, Department of Chemistry and Pharmacy, Friedrich-Alexander-Universität Erlangen-Nürnberg, Erlangen, Germany

7 Department of Anesthesiology and Intensive Care Medicine, Medical Faculty, Otto-von-Guericke-Universität Magdeburg, Magdeburg, Germany

8 Department Systems Physiology of Learning, Leibniz Institute for Neurobiology

9 Center for Behavioral Brain Sciences, Magdeburg, Germany

10 Department of Clinical Psychology, Friedrich Schiller University, Jena, Germany

11 School of Medicine, Université Paris-Saclay, Le Kremlin-Bicêtre, France

12 Department of psychiatry, CHU Bicêtre, APHP, Le Kremlin-Bicêtre, France

13 Inserm, CESP, MOODS team, Le Kremlin-Bicêtre, France

14 Department of psychiatry, CHU Nîmes, France

15 Department of Psychiatry, McGill University, Montreal, Canada

16 Department of Behavioral Neurology, Leibniz Institute for Neurobiology, Magdeburg, Germany

17 Department of Biomedical Magnetic Resonance, Otto von Guericke University, Magdeburg, Germany

18 Systems Neurophysiology, Department of Biology, Darmstadt University of Technology, Darmstadt, Germany

19 Max Planck Institute for Biological Cybernetics, Tübingen, Germany

**Supplementary Methods**

**Magnetic resonance spectroscopy data preprocessing and metabolite fitting**

Spectral data (0.6–4.0 ppm) were analyzed with LCModel (Stephen Provencher, Inc., Oakville, ON, Canada, V6.3.0) using a scanner- and sequence-specific basis set. The basis set included creatine, glutamate (Glu), myo-inositol, lactate, N-acetylaspartate, phosphocholine, taurine, aspartate, gamma-Aminobutyric acid (GABA), glutamine (Gln), glucose, alanine, N-acetyl-aspartyl-glutamate, phosphocreatine, scyllo-inositol, acetate, succinate, phosphorylethanolamine, glutathione, citrate, and glycerophosphocholine. Spectra were excluded based on visual inspection of the fitted curve and objective quality criteria: Cramér Rao lower bounds (CRLB)>20 %, line width of the magnitude signal at FWHM>24 Hz, or signal-to-noise ratio<20. The fitted Glu and Gln concentration levels were normalized to individual non-cerebrospinal fluid (non-CSF: gray matter (GM) and white matter (WM)) volume and used for subsequent analyses. Furthermore, according to the analysis of Evans et al., [(1)](https://www.zotero.org/google-docs/?He8BzJ) and Bednarik et al., [(2)](https://www.zotero.org/google-docs/?TG2WnC), a post-hoc power analysis (power=80 %, alpha=0.05) was employed to estimate the minimal detectable concentration differences for Glu and Gln. For exploratory purposes only and not directed towards answering our primary research question, GABA level changes were analyzed in accordance with the analysis of the primary metabolites of interest, Glu and Gln. Furthermore, the minimal detectable concentration difference was calculated for GABA accordingly.

The 40 % overlap in the group level mask that was used as a rsFC seed for the present analysis refers to an overlap across all participants across both placebo and ketamine arm baselines. Seventy measurements were used to generate a group-level mask. Only baseline measurements were used since MRS voxels for measurements during infusion and 24 h after infusion were placed on the corresponding baseline reference using a vendor-provided automatic voxel positioning technique ensuring the identical voxel placement [(3)](https://www.zotero.org/google-docs/?KfRAsb) per participant. Supplementary Fig. S1 shows examples of the participants with variable shapes of the cingulate cortex and the pericallosal vein located in front of the corpus callosum. This vein had to be circumvented when placing the MRS voxel, leading to a slightly variable position between the participants due to anatomical differences. This problem is usually less apparent when acquiring data using 3 T scanners since these veins are almost undetectable, but becomes noticeable on 7 T images. In order to measure the cingulate area covering predominantly GM and not to compromise the metabolite signals with vein signal, a clean voxel positioning was prioritized over trying to achieve an increased overlap by positioning the voxel less individually with respect to general anatomical landmarks.

**Structural and functional magnetic resonance imaging data preprocessing**

For anatomical MR data preprocessing, an individualized average T1-weighted (T1w) image was calculated from all T1w images (4 images per participant) using CAT12 (CAT12 in SPM12, http://www.neuro.uni-jena.de/cat/). The derived average T1w image was corrected for intensity non-uniformity and used as a T1w reference throughout the workflow for all sessions. Volume-based spatial normalization to the standard space (MNI152NLin2009cAsym) was performed through nonlinear registration with antsRegistration (ANTs 2.2.0), and the normalized skull-stripped T1w-reference was segmented into CSF, WM, and GM. The nonlinear transformation for the spatial normalization was saved and further applied to the pregenual anterior cingulate cortex (pgACC) magnetic resonance spectroscopy (MRS) voxel. The volume of GM and WM in each MRS voxel was extracted and used for the normalization of the fitted Glu and Gln levels in the MRS analysis.

The same preprocessing pipeline was performed for all resting-state functional magnetic resonance imaging (rsfMRI) data (six runs per participant). First, a B0-nonuniformity map was estimated based on a phase-difference map calculated with a dual-echo gradient-recalled echo (GRE) sequence and then co-registered to the generated individual echo-planar imaging (EPI) references in fMRIPrep. Based on the estimated susceptibility distortion, a corrected EPI reference was calculated and co-registered with the anatomical reference. The EPIs were then slice-time corrected. Head-motion parameters in respect to the EPI reference (transformation matrices and six corresponding rotation and translation parameters) were estimated. With the realigned blood oxygenation level dependent (BOLD) images, the noise-related signal was removed using FMRIB’s ICA-based Xnoisifier (FIX; [(4,5)](https://www.zotero.org/google-docs/?wV3Rvz)). Using FIX, noise classification was trained on the baseline scan of 20 participants and then applied to all participants and time points. In order to keep it consistent with existing literature, the head motion as assessed by the mean frame-wise displacement (FD) was calculated before FIX application and compared between sessions. Paired t-tests revealed no significant difference in head motion between time points or treatment arms. Using the denoised EPI images, several confounding time series were calculated based on the preprocessed BOLD: FD, spatial standard deviation of the data after temporal differencing (DVARS), and the mean signals within the CSF and WM [(6)](https://www.zotero.org/google-docs/?N3KN5m) were employed for the nuisance regression for head motion and physiological signals by xcpEngine (version 1.2.2) [(7)](https://www.zotero.org/google-docs/?7ijkHL). Prior to the regression, a temporal filter (0.01-0.08 Hz) was applied to the confound time series and the preprocessed rsfMRI data to avoid the re-introduction of filtered frequencies [(8)](https://www.zotero.org/google-docs/?01elP2).

**Linear mixed effect models**

LME models for both the immediate (including baseline and acute measures as well as both treatments, ketamine and placebo) and for the delayed effects (including baseline and 24h measures as well as both treatments, ketamine and placebo) were conducted. Due to the present study design, the LME model was performed as follows: Treatment+Baseline+(1|Participant). In the rsFC LME models, group maps were FWE-corrected to p<0.05 with an initial threshold of p<0.001 (in the absence of results, the initial threshold was lowered to p<0.005 for exploratory purposes). For the metabolite LME model, statistical thresholds were set at p<0.05.

**Correlation of immediate S-ketamine-induced imaging changes with ketamine (metabolite) plasma concentrations and psychotomimetic effects**

The immediate S-ketamine-induced imaging changes (rsFC changes (during infusion-baseline) in the medial prefrontal cortex (mPFC) and dmPFC) were correlated with the blood plasma levels of ketamine and its metabolites (norketamine (NK) and hydroxynorketamine (HNK)) as well as with the psychotomimetic effects.

**Supplementary results**

**Immediate and delayed GABA level changes induced by S-ketamine**

In addition to Glu and Gln, also GABA level changes were analyzed. We did not see significant immediate or delayed changes in GABA levels following S-ketamine infusion in either treatment arm (for metabolite levels, see Supplementary Table S1). The minimal detectable concentration difference for GABA in this dataset was estimated as 0.32 μmol/g (~21 %).

**Linear mixed effect models results for immediate and delayed S-ketamine-induced rsFC and glutamate changes**

For the rsFC, the treatment effect of the LME model with the baseline as a regressor showed significant clusters in the dorsomedial prefrontal cortex (dmPFC), dorsolateral prefrontal cortex (dlPFC) and orbitofrontal cortex (OFC) (group maps were FWE-corrected to p<0.05 with an initial threshold of p<0.001) for the immediate effects (Supplementary Fig. S2). Furthermore, the LME model conducted for the delayed effects showed a significant cluster in the inferior parietal cortex (IPL) for the treatment contrast (there was no significant cluster for an initial cluster level threshold of p<0.001, but in the light of the exploratory character of the analysis the threshold was lowered to p<0.005) (Supplementary Fig. S3). There were no significant results for the LME models conducted in the spectroscopy modality.

**Results of correlational analysis of acute S-ketamine-induced imaging changes with ketamine (metabolite) plasma concentrations and psychotomimetic effects**

There were no significant correlations of the immediate imaging measures (rsFC changes (during infusion-baseline) in the medial prefrontal cortex (mPFC) and dmPFC) with the psychotomimetic effects (mainscales of the 5-Dimensional Altered States of Consciousness Rating Scale, 5D-ASC [(9)](https://www.zotero.org/google-docs/?L5jr4Y)) (p’s>0.05) or with the acquired blood level concentrations (ketamine, NK and HNK blood plasma levels) (p’s>0.05).

**Supplementary Figure Legends**

**Supplementary Fig. S1:**

PgACC voxel positioning for four participants showing anatomical differences in the cingulate cortex shape and pericallosal veins.

Abbreviation: pgACC=pregenual anterior cingulate cortex.

**Supplementary Fig. S2:**

LME model results for the immediate effects of the pgACC-centered rsFC analysis. For the LME model including S-ketamine and placebo during infusion measurements with baseline as a regressor, four significant clusters were observed: dmPFC (7, 23, 59; pFWEc<0.001), dlPFC1 (-44, 43, 0; pFWEc<0.001), dlPFC2 (-38, 15, 53; pFWEc<0.001), OFC (47, 33, -14; pFWEc<0.001).

Abbreviations: dmPFC=dorsomedial prefrontal cortex; dlPFC=dorsolateral prefrontal cortex; pgACC=pregenual anterior cingulate cortex; OFC=orbitofrontal cortex; rsFC=resting-state functional connectivity.

**Supplementary Fig. S3:**

LME model results for the delayed effects of the pgACC-centered rsFC analysis. For the LME model including S-ketamine and placebo 24 h measurements with baseline as a regressor, one significant cluster was found: IPL (47, -56, 49; pFWEc<0.005).

Abbreviations: IPL=inferior parietal lobe; pgACC=pregenual anterior cingulate cortex; rsFC=resting-state functional connectivity.

**Supplementary Fig. S4:**

Correlations between the adjusted baseline and delayed a) pgACC-IPL rsFC and b) pgACC Glu level changes. Significant negative correlations between the delayed rsFC decrease with the adjusted baseline rsFC between pgACC and left IPL (rp=-0.47; α=0.05, p=0.0053) and between the delayed Glu level increase (24 h after infusion-baseline) and the respective adjusted baseline Glu level (rs=-0.5; α=0.05, p=0.004) in pgACC was observed. Correlations were calculated based on Oldham’s method [(10)](https://www.zotero.org/google-docs/?eywZMV), correlating the pairs (24 h after infusion-baseline) and (24 h after infusion+baseline)/2 (here referred to as adjusted baseline of rsFC or Glu level, respectively).

Abbreviations: Glu=glutamate; pgACC=pregenual anterior cingulate cortex; IPL=inferior parietal lobe; rsFC=resting-state functional connectivity.

**Supplementary Fig. S5**:

Correlation of the delayed rsFC change between the pgACC-IPL with KET48. The delayed rsFC increase (24 h after infusion-baseline) correlated positively with the ketamine plasma concentration acquired 48 min after the start of the infusion (rp=0.33; α=0.05, p=0.06).

Abbreviations: KET48=ketamine plasma concentration acquired 48 min after the start of the infusion; pgACC=pregenual anterior cingulate cortex; rsFC=resting-state functional connectivity.

**Supplementary Figures**

**Supplementary Fig. S1**

**
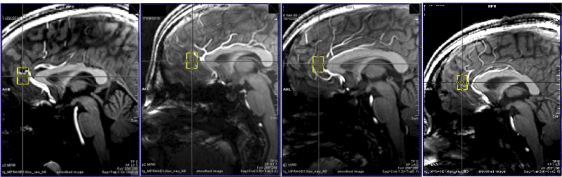
**

**Supplementary Fig. S2**

**
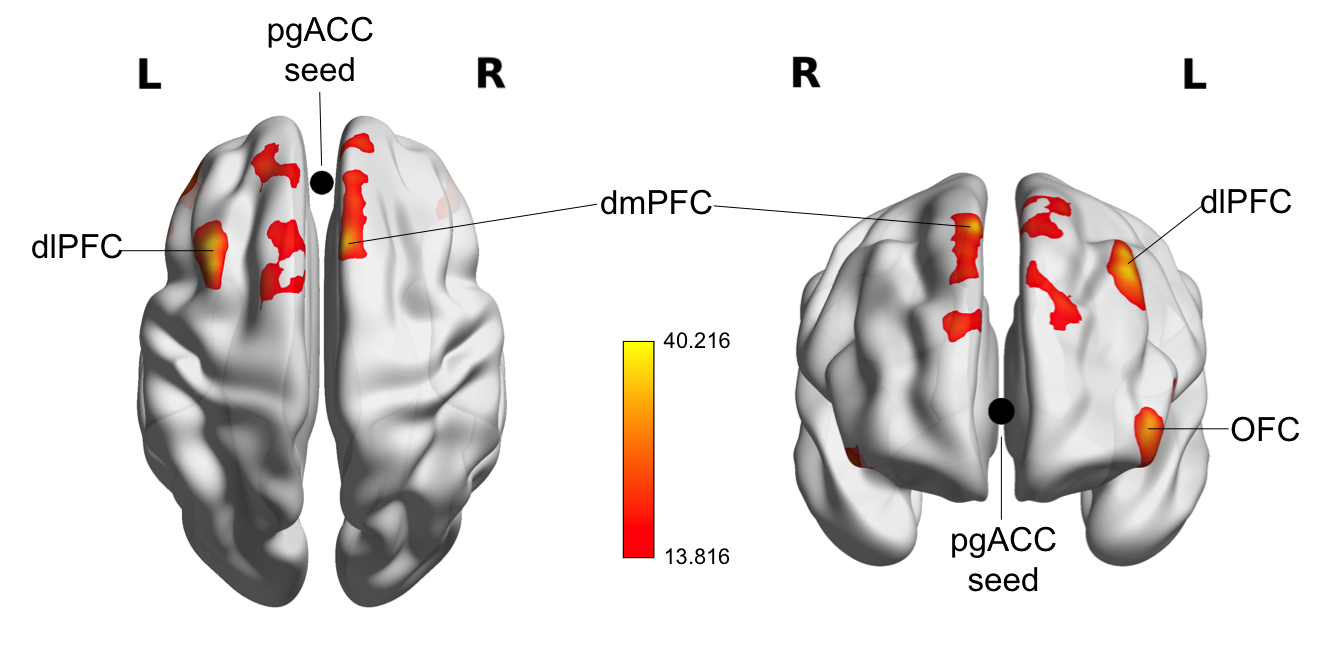
**

**Supplementary Fig. S3**

**
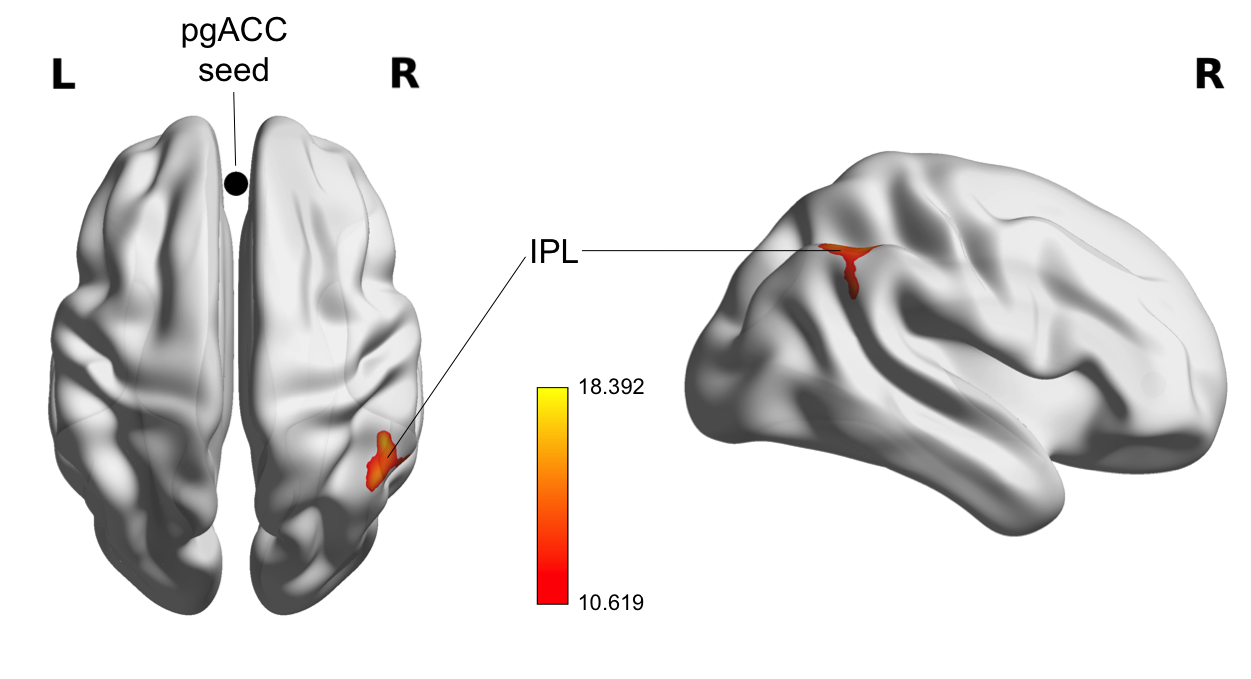
**

**Supplementary Fig. S4**


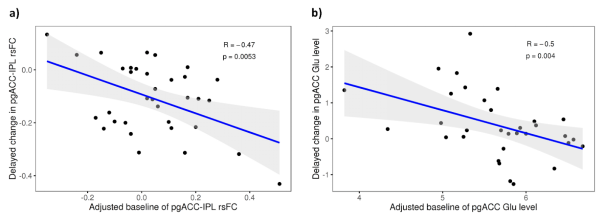


**Supplementary Fig. S5**


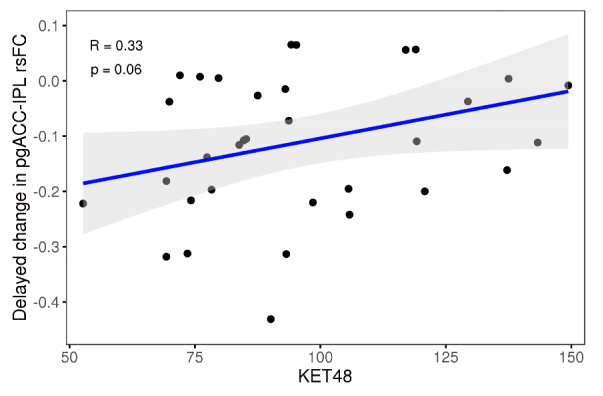


**Supplementary Tables**

**Supplementary Table S1**: Metabolite level and number of included participants for magnetic resonance spectroscopy metabolites per time point and treatment group.

| Metabolite | Group | Baseline | During infusion | 24 h after infusion |
| --- | --- | --- | --- | --- |
| Glu (i.u.) | Placebo | 5.68±0.87 (n=32) | 5.33±0.76 (n=31) | 5.71±0.84 (n=32) |
|  | Ketamine | 5.39±0.97 (n=34) | 5.37±0.90 (n=32) | 5.84±0.59 (n=33) |
| Gln (i.u.) | Placebo | 2.16±0.97 (n=28) | 2.37±0.52 (n=29) | 2.14±0.60 (n=27) |
|  | Ketamine | 2.29±0.70 (n=33) | 2.15±0.67 (n=28) | 2.17±0.36 (n=32) |
| Gln/Glu (i.u.) | Placebo | 0.39±0.18 (n=28) | 0.45±0.14 (n=29) | 0.38±0.12 (n=27) |
|  | Ketamine | 0.45±0.18 (n=33) | 0.41±0.15 (n=28) | 0.37±0.08 (n=32) |
| GABA | Placebo | 1.75±0.59 (n=27) | 1.61±0.35 (n=31) | 1.58±0.43 (n=28) |
|  | Ketamine | 1.49±0.49 (n=32) | 1.60±0.44 (n=30) | 1.60±0.40 (n=31) |

Abbreviations: GABA=gamma-Aminobutyric acid; Gln=glutamine; Glu=glutamate; i.u.=institutional units; n=number of included subjects; pgACC=pregenual anterior cingulate cortex. All values are expressed using mean±SD.

**Supplementary Table S2**: Plasma levels of ketamine and ketamine metabolites and available data points per time point.

| Metabolite | Time point (min after start of infusion) | | |
| --- | --- | --- | --- |
|  | 8 | 13 | 48 |
| Ketamine  concentration (ng/ml) | 49.5±24.06 (n= 31) | 48.4±15.1 (n=31) | 102.2±25.75 (n=35) |
| Norketamine  concentration (ng/ml) | 0.8±0.05 (n=2) | 4.2±2.28 (n=31) | 34.6±10.43 (n=35) |
| Hydroxynorketamine  concentration (ng/ml) | n.q./n.d. (n=0)* | 3.5±1.54 (n=15) | 18.2±6.51 (n=35) |

Abbreviations: n=number of available data points; n.q.=not quantifiable; n.d.=not detectable. All values are expressed using mean±SD. *The concentration of hydroxynorketamine was below the limit of detection (n=11) or the limit of quantification (n=20), respectively, in all samples investigated at this time point.

**Supplementary Table S3**: Changes in the pgACC rsFC during and 24 h after infusion in the ketamine arm (n=34, adjusted α=0.025).

| Seed region | Time point |  | MNI coordinates (mm) | | | k | p |
| --- | --- | --- | --- | --- | --- | --- | --- |
|  |  |  | x | y | z |  |  |
| pgACC | During infusion | mPFC ↑ | 2 | 56 | 24 | 924 | <0.001 |
|  |  | dmPFC ↑ | 14 | 38 | 52 | 338 | 0.005 |
|  | 24 h after infusion | Left IPL ↓ | -44 | -42 | 38 | 239 | 0.018 |
|  |  | Right dlPFC ↓ | 40 | 16 | 38 | 368 | 0.002 |
|  |  | Cerebellum ↓ | 34 | -60 | 32 | 250 | 0.015 |

Abbreviations: dlPFC=dorsolateral prefrontal cortex; dmPFC=dorsomedial prefrontal cortex; k=cluster size; mPFC=medial prefrontal cortex; pgACC=pregenual anterior cingulate cortex; IPL=inferior parietal lobe; n=number of included subjects; rsFC=resting-state functional connectivity. Arrows ↑ and ↓ indicate increased and decreased resting-state functional connectivity towards the seed regions compared to baseline, respectively.

**References**

[1. Evans JW, Lally N, An L, Li N, Nugent AC, Banerjee D, et al. 7T (1)H-MRS in major depressive disorder: a Ketamine Treatment Study. Neuropsychopharmacol Off Publ Am Coll Neuropsychopharmacol. 2018 Aug;43(9):1908–14.](https://www.zotero.org/google-docs/?B2eS1M)

[2. Bednarik P, Spurny B, Silberbauer LR, Svatkova A, Handschuh PA, Reiter B, et al. Effect of Ketamine on Human Neurochemistry in Posterior Cingulate Cortex: A Pilot Magnetic Resonance Spectroscopy Study at 3 Tesla. Front Neurosci. 2021;15:609485.](https://www.zotero.org/google-docs/?B2eS1M)

[3. Dou W, Speck O, Benner T, Kaufmann J, Li M, Zhong K, et al. Automatic voxel positioning for MRS at 7 T. Magma N Y N. 2015 Jun;28(3):259–70.](https://www.zotero.org/google-docs/?B2eS1M)

[4. Griffanti L, Salimi-Khorshidi G, Beckmann CF, Auerbach EJ, Douaud G, Sexton CE, et al. ICA-based artefact removal and accelerated fMRI acquisition for improved resting state network imaging. NeuroImage. 2014 Jul;95:232–47.](https://www.zotero.org/google-docs/?B2eS1M)

[5. Salimi-Khorshidi G, Douaud G, Beckmann CF, Glasser MF, Griffanti L, Smith SM. Automatic denoising of functional MRI data: combining independent component analysis and hierarchical fusion of classifiers. NeuroImage. 2014 Apr;90:449–68.](https://www.zotero.org/google-docs/?B2eS1M)

[6. Power JD, Mitra A, Laumann TO, Snyder AZ, Schlaggar BL, Petersen SE. Methods to detect, characterize, and remove motion artifact in resting state fMRI. NeuroImage. 2014 Jan;84:320–41.](https://www.zotero.org/google-docs/?B2eS1M)

[7. Ciric R, Wolf DH, Power JD, Roalf DR, Baum GL, Ruparel K, et al. Benchmarking of participant-level confound regression strategies for the control of motion artifact in studies of functional connectivity. NeuroImage. 2017 Jul;154:174–87.](https://www.zotero.org/google-docs/?B2eS1M)

[8. Hallquist MN, Hwang K, Luna B. The nuisance of nuisance regression: spectral misspecification in a common approach to resting-state fMRI preprocessing reintroduces noise and obscures functional connectivity. NeuroImage. 2013 Nov;82:208–25.](https://www.zotero.org/google-docs/?B2eS1M)

[9. Dittrich A. The standardized psychometric assessment of altered states of consciousness (ASCs) in humans. Vol. 31, Pharmacopsychiatry. Germany: Georg Thieme Verlag KG; 1998. p. 80–4.](https://www.zotero.org/google-docs/?B2eS1M)

[10. Oldham PD. A note on the analysis of repeated measurements of the same subjects. J Chronic Dis. 1962 Oct;15:969–77.](https://www.zotero.org/google-docs/?B2eS1M)
